# Physiological and Somatic Principal Components Determining VO_2_max in the Annual Training Cycle of Endurance Athletes

**DOI:** 10.3390/ijerph19073951

**Published:** 2022-03-26

**Authors:** Natalia Grzebisz-Zatońska, Stanisław Poprzęcki, Arkadiusz Stanula, Ewa Sadowska-Krępa, Dagmara Gerasimuk

**Affiliations:** 1 Faculty of Cosmetology, Warsaw College of Engineering and Health, Bitwy Warszawskiej 1920 Street 18, 02-366 Warsaw, Poland; 2 Institute of Sport Sciences, Jerzy Kukuczka Academy of Physical Education in Katowice, Mikołowska Street 72a, 40-065 Katowice, Poland; s.poprzecki@awf.katowice.pl (S.P.); a.stanula@awf.katowice.pl (A.S.); e.sadowska-krepa@awf.katowice.pl (E.S.-K.); d.gerasimuk@awf.katowice.pl (D.G.)

**Keywords:** endurance effort, cycling, training control, physical fitness, VO_2_max, physiological determinants

## Abstract

The purpose of the study was to assess the impact of training on the physiological variables achieved during the test effort in the macrocycle of road cyclists and their use in the maximal oxygen uptake (VO_2_max) prediction at individual training stages in the VO_2_max test. Nine well-trained male cyclists (age 25.6 ± 5.2 years and body weight 72.4 ± 7.35 kg) participated in the study and each phase of the macrocycle was followed by a time to exhaustion test (TTE) on the bicycle ergometer. The research showed that training loads significantly influence the maximum power (PPO), ventilation (VE) in the preparatory period (T1), time of the test (TTmax) at the start of the competition period (T2), percentage of body fat in total body weight (%FAT) and skeletal muscle mass (MMS) during the competition period (T3). Of the 16 variables taken for the analysis of the principal components (PC), the regression model determined one principal variable responsible for VO_2_max in the training macrocycle of cyclists, the relative value of maximum power (PPO_RV_) and the accompanying variables in individual periods: breathing frequency (BF), delta blood lactate concentration (ΔLA), body fat (FAT) and MMS. Determining PC influencing the exercise capacity can be crucial in achieving the intended goals by athletes. Monitoring these indicators can help protect the health of professional athletes and provide guidelines in the training process, stimulate the body properly while protecting against overtraining.

## 1. Introduction

The purpose of controlling the effects of training loads during the preparatory season is to obtain information about the relationship between the size of the loads and the body’s response to them, as well as the effectiveness of the training program. Adaptation to the effort is evidenced by the difference between the amount of training load and the fatigue process [1]. In various sports, physical performance largely depends on the integrated status of the various physiological mechanisms. The most important of them influencing endurance in cyclists is high aerobic capacity during maximal exercise, as well as the efficiency of the circulatory and respiratory systems [2]. The maximal oxygen uptake (VO_2_max) is a well-known marker of cardiorespiratory fitness and is associated with good health outcomes [3]. In addition to the “protective” health effects attributed to cardiorespiratory fitness [4], VO_2_max is also the primary determinant of endurance capacity, explaining about 20–60% of the variability in performance with different modes and distances, which can be achieved by athletes in combination with other determinants of maximum endurance capacity [5,6].

During the time to exhaustion test (TTE) in which VO_2_max is determined, changes in the values of variables such as power (PO), heart rate (HR), oxygen uptake-VO_2_, and time of the test (TTmax) are frequently assessed indicators [7]. Professional cyclists are characterized by high physical efficiency, over 60 mL/kg/min, and generating high PO-over 400 W during TTE [8]. During the preparation for the racing season, the specific training goals at various stages of the macrocycle are differentiated, which implies the use of appropriate physiological control [1,9]. In monitoring the training of cyclists, physiological parameters such as VO_2_, HR, PO and their interrelationship as a result of cardiorespiratory fitness were most often assessed. There is currently a lack of research that follows year-round training of professional athletes and identifies the principal components (PC) of the physiological and somatic group that affect the VO_2_max. The results of this monitoring method can be used by the training staff in assessing the effectiveness of training and exercise capacity.

The study aimed to assess the impact of training on the physiological variables achieved during TTE in the macrocycle of road cyclists and their use in VO_2_max prediction at individual training stages. The hypothesis was that endurance training affects the VO_2_max in the macrocycle of this group, and the physiological (e.g., VO_2_max, VE, HR) and somatic variables (e.g., BMI—Body Mass Index; FAT—body fat mass) can be used in VO_2_max prediction at individual training stages. Determining PC influencing exercise capacity and training can be of key importance in achieving goals by athletes. It can be used by coaches, physiologists and sports doctors during the preparation of competitors for the main starts and in the prevention of overtraining syndrome.

## 2. Materials and Methods

### 2.1. Participants

Nine well-trained road cyclists participated in the study (the study recruited athletes from sports associations in Poland), with a minimum of 5 years of training experience and a VO_2_max of approx. 5.0 L/min (approx. 65 mL/kg/min), aged 25.6 ± 5.2 years and body height 181.0 ± 5.6 cm. Other data are presented in Table 1. During the macrocycle, the athletes implemented an individual training program (prepared by a professional trainer) based on the concept of linear periodization. During the preparations, four TTE were performed on a bicycle ergometer: T0 (TRAN: after transition period—27–30 November 2014 and 8–10 December 2014), T1 (PREP—preparatory period—2–5 February 2015), T2 (COMP I—start of competition period—14–16 April 2015), T3 (COMP II—during competition period—1–4 July 2015). The workload in hours was for the subsequent periods 157, 144, and 189, respectively. Training included developing endurance, strength and speed. Comprehensive, specialized and targeted workouts were performed that were typical of cycling. The participants signed informed consent about the procedures and possible injuries that could result from the study. The study was approved by the Local Bioethical Committee University Ethics Committee decision Nr. 2/2014 and conducted by the Declaration of Helsinki of the World Medical Association.

The training loads in each stage of the study were individually recorded by the competitors using a heart rate monitor (Garmin, Olathe, KS, USA). The authors of the work did not participate in the planning and corrections of training tasks in PREP. Table 2 shows the average training load in individual periods of the training macrocycle, taking into account the percentage of work in the zone of aerobic, mixed and anaerobic metabolic changes. The volume of training in hours in individual test stages and their intensity in individual exercise zones are presented in Table 2.

### 2.2. VO_2_max Test and the Body Composition Measurement

TTE was performed four times: in TRAN, PREP, COMP I and COMP II. Before the VO_2_max test, body height (cm) was assessed during the T0 stage, with an anthropometer accurate to 0.5 cm (Vitako & Vbody-Nutrition, Health & Sport Equipment Vitako Sp. z o.o., Poland, Warsaw). Then, before each test, the body weight (kg) was determined with an accuracy of 100 g. The body composition, BMI and percentage of body fat in total body weight (FAT%) were determined using the electrical impedance method using the In-Body 570 analyzer (In Body Ltd., Seoul, Korea). All VO_2_max tests were performed on the same ergometer (LODE Excalibur Sport, Lode BV, Groningen, The Netherlands), which was individually adapted to the rider. The ergometer was coupled to a gas analyzer-MetaLyzer-3B-R2 (CORTEX Biophysics GmbH, Leipzig, Germany). TTE was performed in similar environmental conditions—19–21 degrees of Celsius, 40–50% relative humidity, each time in the morning. Standard VO_2_max test was performed with an initial load of 40 W, then the load was increased by 40 W every 3 min until the cadence could not be maintained >70 rpm. 

During TTE, the following were recorded: HR with the Sport Tester RS 800 from Polar Inc., in combination with an air-gas analyzer (Polar Inc., Kempele, Finland). In addition, the following were recorded: VO_2_max (mL/kg/min) and (L/min); absolute and relative values of maximum power—PPO (W) and PPO_RV_ (W/kg); maximum minute ventilation VEmax (L/min); index—PPO/VO_2_max; index VO_2_max/HRmax; maximum heart rate—HRmax; maximum test duration—TTmax (s); breathing frequency/respiratory rate—BF (1/min); respiratory exchange rate—RER (VCO_2_/VO_2_); volume CO_2_ (VCO_2_); and increase in blood lactate concentration during exercise ±∆LA (mmol/L). Fingertip capillary blood samples for LA assessment (Biosen C line Clinic, EKF-diagnostic GmbH, Barleben, Germany) were collected just before TTE (while standing), at the end of each exercise load (every 3 min), as well as 4–5 min after TTE (while seating). Number of blood lactate measurements during exercise depending on the time of TTE. In addition, somatic variables were recorded: body mass—BM (kg); %FAT; FAT (kg); BMI; skeletal muscle mass—MMS (kg); lean body mass—FFM (kg); and total body water content—TBW (L).

The HRmax and VO_2_max values were defined as the highest heart rate and oxygen uptake values obtained in TTE. The value of PPO was determined for the highest last load in the progressive test by the software of the MetaLyzer 3B-R2 gas analyzer (30 s). The lactate threshold (LT) was defined as the intensity of exercise at which there was an increase in lactate concentration above baseline (the exercise intensity corresponding to a blood lactate concentration of 2.5 mmol × L^−1^). The LT was determined by the D-max method. The anaerobic threshold (AT) was defined as the exercise intensity above which blood lactate begins to rise sharply (the exercise intensity corresponding to a blood lactate concentration of 4 mmol × L^−1^). It is defined the same as the onset of blood lactate accumulation (OBLA). The individual anaerobic threshold (IAT), also defined as the maximal lactate steady state, was explained as the maximal exercise intensity that can be continuous without a rise in the blood lactate concentration. The lactate inflection point was defined as the exercise intensity at which the blood lactate concentration begins to increase significantly and was created by drawing tangents [10,11,12]. In addition, athletes were instructed to refrain from vigorous exercise on the day before TTE, and similar eating habits were maintained before each measurement. 

### 2.3. Statistical Analysis

Data are presented as average values and standard deviation (m ± SD). The normality of data distributions was tested using the Shapiro–Wilk W-test. For multiple comparisons between the mean values of the physiological variables of the VO_2_max test and the somatic features, general linear models (GLMs) with repeated measurements for dependent groups using Bonferroni’s post hoc analysis were used. The measurements were repeated against the variable “test” [(T0), (T1), (T2) and (T3)]. The physiological variables and body composition (+/−∆%), in the training macrocycle of cyclists with the base values T0, T1 − T0, T2 − T0 and T3 − T0, were calculated according to the formula ∆% = (T1, T2, T3 − T0)/T0 × 100. To reduce the data to a set of PC, principal component analysis (PCA) was used. Each PC contains a set of correlated variables. At the same time, PC are not correlated with each other. This causes each major component to provide separate information. Before performing PCA, the Pearson correlation matrix was visually inspected to determine the factorability of the data for PCA. The number of significant factors was determined by the Kaiser–Guttman criterion, which retains PC with eigenvalues of 1.0 or greater. At a later stage of the analysis, multiple regression was used, during which for the estimation of VO_2_max at each stage of the study, independent variables were components distinguished as a result of PCA. Statistical significance was set at *p* ≤ 0.05. Statistical calculations were performed using Statistica 13.3 (TIBCO Software Inc., Palo Alto, CA, USA) for MS Windows 10. 

## 3. Results

Table 3 presents the physiological variables of the VO_2_max test and the somatic variables of road cyclists in four periods of the training macrocycle. In the group of physiological variables only for PPO (W) and VEmax (L/min), there was a significant increase in the value in the T1 compared to T0 (436.7 ± 31.45 vs. 404.3 ± 33.67; *p* ≤ 0.05 and 180.3 ± 24.11 vs. 163.7 ± 17.81; *p* ≤ 0.01, respectively). In the group of somatic variables, a significant increase was noted in the T3 for MMS (kg) (37.4 ± 3.53 vs. 36.5 ± 3.61; *p* ≤ 0.05) and a decrease in FAT (%) (9.2 ± 2.13 vs. 10.7 ± 2.78; *p* ≤ 0.05).

**Figure 1:**
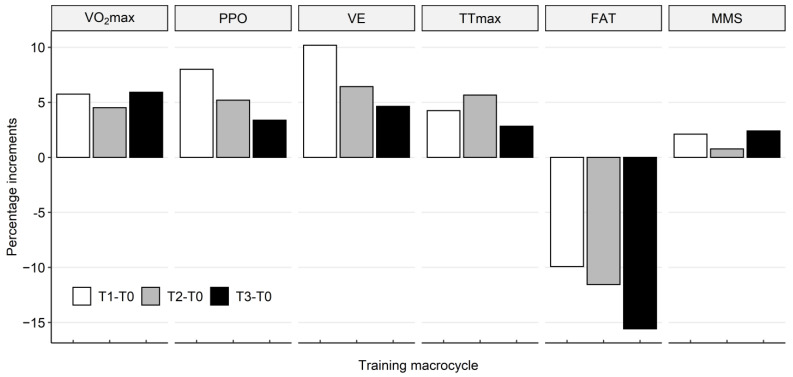
Percentage increases in the levels of physiological and somatic variables of cyclists in individual research periods (+/−∆%), in the training macrocycle of cyclists with the base values T0: T1 − T0, T2 − T0 and T3 − T0. Abbreviations: VO_2_max—the maximal oxygen uptake; PPO—absolute value of maximum power; VE—ventilation; TTmax—maximum test duration; FAT—current body fat mass; MMS—skeletal muscle mass.

Figure 1 shows the percentage increments of physiological variables (VO_2_max, PPO, VE, TTmax) and somatic variables (FAT and MMS) relative to the base level (T0), which react significantly to training loads in individual research periods. The highest increase (10.2%) is observed for VE (L/min) in T1 and FAT (%) (15.6%) in T3.

### Principal Component Analysis 

Table 4 presents the results of the analysis of PCA for individual training periods described based on 16 physiological variables and body composition. PCA shows that in T0, the first four PC explain together about 95% of the variability (50.37, 20.01, 16.75 and 8.14 for PC1, PC2, PC3 and PC4, respectively). In T1, the first four PC explain together about 92% of the variability (55.78, 16.54, 13.64 and 6.32 for PC1, PC2, PC3 and PC4, respectively). In T2, the first three PC explain about 85% of the variability, and their percentage contribution to the explanation of the variability of this PC is, respectively: 38.25, 23.45 and 15.47 for PC1, PC2 and PC3. The analysis carried out for T3 showed that the first three PC together explain about 85% of the variance (54.68, 19.46 and 11.21 for PC1, PC2 and PC3, respectively). PC1 in each macrocycle period contained the most numerous factor charge (Table 4). 

Based on PCA for individual training periods, multiple regression models were built, in which the dependent variable was the VO_2_max result, and PCs were independent variables. The results of regression analysis are presented in Table 5.

Constructed models of multiple regression, in which the dependent variable was VO_2_max and independent variables were PCs, in three cases were statistically significant (Model 1, 3, 4) and in one non-essential (Model 2). Table 5 shows that a significant independent variable PC2 occurred in three models. Thus, this isolated PC generally explained the VO_2_max dependent variable.

## 4. Discussion

The purpose of the study was to assess the impact of training on the physiological variables achieved during the test effort in the macrocycle of road cyclists and their use in the maximal oxygen uptake (VO_2_max) prediction at individual training stages in the VO_2_max test.

The results of this study did not show a significant effect of cycling training on the value of VO_2_max and HRmax. The results are consistent with the works of other authors, for example, Lucia et al. [13,14] and Sassi et al. [15,16]. They showed that the components of the VO_2_max test are not sensitive to training-induced changes in the case of their high baseline values in the macrocycle, as is often found in professional cyclists with a capacity corresponding to a VO_2_max of 70–85 mL/kg/min. In some studies, several percent increases in VO_2_max were observed in PREP. However, the magnitude of these changes depended on the baseline value. In general, the higher the performance level of the athletes, the more stable the VO_2_max value was throughout the season [8].

Despite the suggested limited training capacity for VO_2_max [17], many different training methods are used to support the adaptation of this parameter, such as repetitive high-intensity exercise or continuous endurance exercise, which usually produces moderate effects [18]. However, it was found that training loads significantly differentiated the level of PPO (W) (*p* = 0.016). The greatest increase in PPO was observed in T1, i.e., at the peak of PREP, by 8.21% and 7.82%, respectively. In T1, the average PPO value was 436.67 ± 31.45 W. The reason could probably be the increased energy value of meals during the holiday season and stationary training, mainly strength training. After this period, a tendency to lower PPO of 3.39% was observed. These results also indicate that low- and moderate-intensity training with resistance training (which dominated in PREP) contributed to the increase in endurance capacity. Adding high-intensity training reduces capacity, but improves adaptation to competition efforts (COMPI). Similar conclusions were presented in the article by Saw et al. [19]. At the same time, it underlines the importance of endurance capabilities and their role in achieving the goal during the competition period [20]. Increasing endurance capabilities is crucial for results, for example in long-lasting races [21]. Moreover, during the annual training cycle, VEmax was significantly differentiated (*p* = 0.005). Significant increase in VEmax during T1 (*p* = 0.003) about T0, after which the value slightly decreased by 4.76% about T0. This increase is beneficial for the athlete and shows that he is adapting to the effort at the beginning. High values of this parameter characterize well-trained athletes and can determine the VO_2_max [22,23,24,25]. The training significantly influenced TTmax *(p* = 0.020). A significant increase (5.98%) was recorded in T2 (*p* = 0.018), i.e., at the beginning of the competition period. These data can be applied to the studies by Costa et al. [26] who showed that TTmax of the second run was significantly longer compared to the first run.

Training significantly differentiated the FAT and MMS during COMPII. The increase in the MMS and decrease in FAT value are beneficial for the athlete. It affects more power over a longer period, which contributes to better sports performance. In the research of Sanada et al., it was indicated that MMS was closely associated with the absolute VO_2_max, independently of BM or FFM [27]. A larger 2 kg of FFM (about 1 kg of MMS) also contributes to the change in VO_2_max (increase by 200 mL/min) [28]. In addition, FAT significantly affects VO_2_max [29,30]. The FAT (Lee et al. (2002)) in the cycling group was 7.9 ± 1.8% and was lower than the lowest values recorded in our studies (9.17 ± 2.13) [31]. According to Jeukendrup et al. (2010), male athletes should have body fat levels in the range of 5–10% [32]. This indicates that further downward trends in FAT to the lower recommended limits may affect the increase in VO_2_max. On the other hand, a reduction in FAT below 4%, however, can affect the body’s regenerative capacity and adversely affect the immune system [33]. The key in the training process is therefore to optimize FAT, but to the lower reference values without exceeding them.

### 4.1. Principal Components

PCA analysis in T1 and T2 identified four PCs and in T3 and T4 three factors each. Each time PC1 explained the highest percentage of variance. In T0, it explained 50.37% variance and included five physiological variables and four somatic variables. In T1, PC1 explained 55.75% variance and included six significantly related physiological variables and 4 somatic variables. In T2, it explained 38.25% of the variance and included two physiological variables and four somatic variables. In T3, PC1 explained a 54.65% variance and contained five strongly related physiological variables and six somatic variables (Table 4).

The results of the regression analysis carried out for the estimation of VO_2_max in T0 allow us to conclude that the obtained model taking into account the three main components (PC2, PC3 and PC4) explains 72% (R^2^ adj.) of the variance of the VO_2_max variable. The average difference between the actual values of the dependent variable and the values predicted by the model was 2.06 (SEE). The value of statistic F (7.73) and the corresponding probability level *p* (*p* < 0.05) confirm the statistical significance of the model. Based on the presented model, it can be estimated that if the value for PC2 increases by one unit, with PC3 and PC4 values unchanged, the VO_2_max value will increase by 0.41 mL/kg/min. In T1, the model turned out to be statistically insignificant (F_(3.5)_ = 2.36; *p* = 0.19). The structural parameters for this model that includes all the main components are presented in Table 5.

The results of the regression analysis taking into account the variables obtained in T2 gave rise to the construction of a statistically significant model (F_(2.7)_ = 6.22; *p* < 0.05) taking into account the two components PC1 and PC2, with the help of which it is possible to explain 54% of the variance of the VO_2_max variable. Based on this model, it can be concluded that reducing the value of PC2 by one unit, with the value of PC1 unchanged, will increase the VO_2_max value by 1.42 mL/kg/min. The regression model built based on the main components distinguished based on variables obtained during the measurements in T3 explains 73% of the variance of the VO_2_max variable. The average difference between the actual values of the dependent variable and the values predicted by the model was 0.88 (SEE). This model takes into account only one variable (PC2), based on which it can be concluded that when the value of the variable PC2 is reduced by one unit, then the VO_2_max value increases by 0.83 mL/kg/min. The value of the F statistic (19.46) and the corresponding probability level *p* (*p* < 0.01) confirm the statistical significance of the model. 

Three statistically significant regression models—Model 1, 3 and 4—significantly explain the VO_2_max dependent variable were distinguished. Among the independent factors, PC2 (Table 5) appeared most often. In T0 it contained the physiological variables PPO_RV_, BF, ΔLA, in T2 it contained the physiological variables PPO_RV_, HRmax and the somatic FAT and FAT% and MMS. In T3, it contained only the physiological variables—PPO_RV_ and VE. Of the 16 variables taken for the analysis of the main factors, the regression model determined one main variable responsible for the oxygen ceiling in the cycling training macrocycle, PPO_RV_, and the accompanying variables in individual periods: BF, ΔLA, FAT and MMS.

Mathematical models can be used to describe and support the prediction of the effect of training on performance. Current models try to describe the impact of a single or series of exercises on the performance of a specific task on a given day as well as in a season. These models suggest that each training increases efficiency and induces a fatigue response in the body [34]. Modeling in swimming training was also used by Avalos et al. [35]. Their study confirmed that in the group of swimmers the mixed model can be use as a basis for the long-term training program because it showed a significant relationship between training and results for the group as well as for individual athletes. In contrast, McLaughlin et al. [36] proposed variables related to endurance VO_2_max, percent of VO_2_max on lactate threshold (%VO_2_maxLT) and running economy as predictors of long-distance runners. The last indicator can be compared to the achievable power in our study. Statistical analysis was performed using the three-stepwise regression model. The best predictor in McLaughlin et al.’s research was VO_2_max, representing 94.4% of the total variance. PCA is widely used by other authors [37,38] to assess the impact of socio-cultural conditions on cyclists’ performance or to determine VO_2_max predictors based on self-reported training loads.

Research shows a relationship between PPO, VO_2_ and HR [30]. This variable can also be used to evaluate the maximum exercise capacity of athletes. The maximum power that is achieved during the test shows a lower measurement error and a coefficient of variation [39]. In our research, the main variable responsible for the oxygen ceiling in the cycling macrocycle was PPO_RV_ (W/kg), which confirms earlier reports. However, not BM, but the share of its components—adipose tissue and MMS, which were the accompanying variables for VO_2_max in individual periods—are of greater importance.

Research has shown that peak power may be due to higher levels of slow-twitch fibers. They are formed in the body of athletes in response to training [40]. In our studies, we did not check changes in fiber type, but we showed that MMS is an important factor in stimulating VO_2_max. A cyclist’s higher PPO may be influenced by the pedal pressure technique, especially on the downstroke [40]. Cycling economy (CE) and gross mechanical efficiency (GE) could compensate for a relatively low VO_2_max in professional cyclists, which emphasizes the importance of MMS [41]. The generated power, which translates into the VO_2_max parameter, depends on the cross-section of the muscles. The sustainable percentage of VO_2_max and CE can be improved by greater use of heavy, resistance training [42]. Additionally, a decrease in FAT can improve exercise capacity [43,44,45].

In studies on endurance athletes, it has been shown that a decrease in FAT affects the improvement of VO_2_max [46]. The components of lung ventilation, the values of which have significantly changed in the annual training cycle, are BF and their capacity. In our research, BF was one of the main determinants of VO_2_max. Breathing efficiency is influenced by, among others, breathing techniques or muscles involved in the biomechanics of this process (diaphragm, external and internal costal muscles, thoracic smaller, sternocleidomastoid and others) [47]. Studies in a group of cyclists indicated that BF is a valid and reliable marker of the anaerobic threshold. Carey et al. [48] tested fifteen professional male cyclists and proved that it is better to use VE and VE/VO_2_ methods than the respiratory rate method (RR) in the assessment of changes in AT in well-trained athletes. The reason for this is the lower standard error of measurement and coefficient of variability. However, our results did not coincide with some of the available ones. According to Nalbandian et al. [49], BF affects some respiratory gas exchange variables but does not influence VO_2_max and endurance performance. We find that greater oxygen availability (also due to an increase in respiratory rate) determines a better metabolism of slow-twitch fibers, which is crucial for cyclists. 

An important major component that was not assessed in our study, but may affect LA, could be MCT1 mRNA. It enables a better removal of lactate and hydrogen ions, which has the effect of extending the duration of exercise [50]. These applications were confirmed in another study [2] and in our study, where a significant decrease in ∆LA during TTE was not noted but was the accompanying variable that determines VO_2_max in individual periods. This may indicate, along with longer effort times, efficient adaptive systems, an increase in the share of aerobic efforts and higher exercise capacity in cyclists [51].

### 4.2. Limitations to the Study

An important principal component disturbing the interpretation of the results of this study was the individualization of training loads causing various physiological responses (although recommended in training), uneven duration of periods between tests, seasonal changes in environmental conditions and a small number of subjects. In addition, the registration of training loads by athletes and the authors’ failure to participate in the planning and course of training. In the future, it would be important to increase the number of cases and continue research with a variety of subjects. Joining the research group of other athletes, including women, with similar exercise capacity would also be a valuable enrichment of this research.

## 5. Conclusions

During the cyclists’ annual training cycle, the absolute value of maximum power, ventilation in the preparatory period, maximum test duration at the start of the competition period and percentage of body fat in total body weight and skeletal muscle mass (MMS) during the competition period change significantly, but not maximal oxygen uptake (VO_2_max). The regression model determined one principal component analysis (PC) responsible for the maximum oxygen uptake, the relative value of maximum power and the accompanying variables in individual periods: breathing frequency, delta blood lactate concentration, body fat and MMS. Monitoring these factors can help protect the health of professional athletes and provide guidelines in the training process, stimulate the body properly and avoid overtraining syndrome. Diagnostics in athletes is crucial in this process. The methodology and test results can be used as a reference to the assessment of changes in the annual training cycle of professional endurance athletes and the determination of the principal components influencing VO_2_max.

## Figures and Tables

**Table 1:** Somatic and functional variables in the annual training cycle of cyclists. Data are presented as mean ± standard deviation.

Variables/Periods Test	TRAN; T0	PREP; T1	COMP I; T2	COMP II; T3
BM (kg)	72.41 ± 7.35	72.63 ± 7.17	72.40 ± 7.00	71.96 ± 6.31
BMI (kg/m^2^)	21.94 ± 1.49	22.02 ± 1.30	21.91 ± 1.29	21.79 ± 1.27
FAT (kg)	7.84 ± 2.51	7.07 ± 2.58	6.94 ± 2.15	6.62 ± 1.74
FAT (%)	10.73 ± 2.78	9.58 ± 3.04	9.50 ± 2.53	9.17 ± 2.13 *

Abbreviations: TRAN—end of the transition period, T0 test; PREP—preparatory period, T1 test; COMP I—start of the competition period, T2 test; COMP II—during the competition period, T3 test; BM—body mass; BMI—Body Mass Index; FAT (kg)— current body fat mass; FAT (%)—the percentage of body fat in total body weight; * statistically significant differences about T0, *p* ≤ 0.05.

**Table 2:** The monthly average values of training loads (volume and intensity, nature of effort, training strategies, nature of cycling training, aerobic and anaerobic training protocol).

Training Period/Test	Month	Training Volume (h)	Training Volume (km)	Training Intensity (% of HRmax)		
				Aerobic	Aerobic– Anaerobic Zone	Anaerobic Zone
TRAN (T0)	November	49.0 ± 3.86	1183.0 ± 82.70	94	4	2
PREP (T1)	December	60.2 ± 6.29	1564.3 ± 116.26	90	6	4
	January	47.3 ± 5.05	1433.2 ± 78.75	70	25	5
	February	71.9 ± 7.90	2001.6 ± 244.02	65	27	8
	March	72.1 ± 8.09	2101.2 ± 185.06	62	23	15
COMP I (T2)	April	76.0 ± 7.55	2158.0 ± 103.51	60	20	20
	May	61.9 ± 3.08	2213.0 ± 113.00	55	25	20
COMP II (T3)	June	50.9 ± 3.52	2097.6 ± 117.48	50	25	25

Abbreviations: TRAN—end of the transition period, T0 test; PREP—preparatory period, T1 test; COMP I—start of the competition period, T2 test; COMP II—during the competition period, T3 test; HRmax—maximal heart rate.

**Table 3:** Selected physiological variables of the VO_2_max test and body composition of road cyclists in four periods of the training macrocycle (T0, T1, T2, T3). Data are presented as mean ± standard deviation.

Variable/Periods	TRAN; T0	PREP; T1	COMP I; T2	COMP II; T3
Physiological variables of VO_2_max test				
VO_2_max (mL/kg/min)	65.8 ± 3.87	69.6 ± 4.10	68.7 ± 4.60	69.7 ± 1.58
PPO (W)	404.3 ± 33.67	436.7 * ± 31.45	425.3 ± 28.8	418 ± 42.67
PPO_RV_ (W/kg)	5.6 ± 0.35	6.0 ± 0.30	6.4 ± 1.40	5.8 ± 0.36
VEmax (L/min)	163.7 ± 17.81	180.3 ** ± 24.11	174.2 ± 17.96	171.3 ± 20.88
BF (1/min)	54.4 ± 9.31	56.7 ± 8.51	54.2 ± 10.48	55.0 ± 6.09
HRmax (bpm)	192.6 ± 6.23	192.2 ± 8.58	191.2 ± 6.46	188.8 ± 8.80
RER (VCO_2_/VO_2_)	1.1 ± 0.05	1.1 ± 0.06	1.2 ± 0.06	1.1 ± 0.05
VCO_2_ (L/min)	5.2 ± 0.40	5.6 ± 0.53	5.5 ± 0.56	5.5 ± 0.55
Somatic variables				
BM (kg)	72.4 ± 7.35	72.6 ± 7.17	72.4 ± 70	72.0 ± 6.31
FAT (kg)	7.8 ± 2.51	7.1 ± 2.58	6.9 ± 2.15	6.6 ± 1.74
FAT (%)	10.7 ± 2.78	9.6 ± 3.04	9.5 ± 2.53	9.2 * ± 2.13
BMI (kg/m^2^)	21.9 ± 1.49	22.0 ± 1.3	21.9 ± 1.29	21.8 ± 1.27
MMS (kg)	36.5 ± 3.61	37.3 ± 3.31	36.8 ± 3.19	37.4 * ± 3.53
FFM (kg)	64.6 ± 6.12	65.7 ± 5.47	64.8 ± 5.34	69.7 ± 1.58

Abbreviations: TRAN—end of the transition period, T0 test; PREP—preparatory period, T1 test; COMP I—start of the competition period, T2 test; COMP II—during the competition period, T3 test; VO_2_max—the maximal oxygen uptake; PPO—absolute value of maximum power; PPORV—the relative value of maximum power; VEmax—maximum minute ventilation; BF—breathing frequency; HRmax—maximal heart rate; RER—respiratory exchange rate; VCO_2_—volume CO_2_; BM—body mass; FAT (kg)— current body fat mass; FAT (%)—percentage of body fat in total body weight; BMI—body mass index; MMS—skeletal muscle mass; FFM—free fat mass; * significant differences from the baseline (T0); * *p* ≤ 0.05 and ** *p* ≤ 0.01.

**Table 4:** Results of the principal component analysis (PCA) for individual periods of training. Table explains the variance and structure of factor changes for 16 features and isolated principal components (PC). In bold, the correlation values of the characteristics most strongly associated with the isolated factors are marked.

**Variables/Explained Variance (%)**	**T0**				**T1**				**T2**			**T3**		
	PC 1	PC 2	PC 3	PC 4	PC 1	PC 2	PC 3	PC 4	PC 1	PC 2	PC 3	PC 1	PC 2	PC 3
	50.37	20.01	16.75	8.14	55.78	16.54	13.64	6.32	38.25	23.45	15.47	54.68	19.46	11.21
PPO	**0.87**	0.39	−0.02	0.27	**−0.92**	0.27	0.22	0.03	**0.75**	0.40	0.01	**−0.90**	−0.32	0.05
PPO_RV_	−0.46	**0.77**	0.06	0.41	**0.72**	0.51	0.29	−0.05	0.32	**0.80**	−0.33	−0.19	**−0.90**	0.00
VEmax	**0.70**	0.55	0.39	−0.20	**−0.67**	0.35	−0.54	−0.24	0.46	0.09	**0.71**	**−0.79**	0.12	0.40
BF	−0.12	**0.59**	0.43	−0.44	0.28	0.26	−0.67	−0.34	−0.37	0.15	**0.71**	0.27	**0.39**	0.37
HRmax	−0.32	−0.58	**0.71**	0.06	0.41	**−0.74**	0.44	−0.06	−0.28	**−0.79**	−0.11	−0.20	−0.46	−0.80
TTmax	**0.86**	0.41	0.05	0.23	**−0.92**	0.12	0.25	0.23	**0.90**	0.21	−0.04	**−0.90**	−0.25	0.03
RER	−0.46	0.19	−0.03	**−0.80**	0.34	−0.27	−0.38	**0.79**	−0.41	0.38	**0.46**	−0.05	**−0.74**	−0.25
VCO_2_	**0.89**	0.34	0.22	−0.06	**−0.89**	−0.05	0.11	0.14	0.42	−0.24	**0.69**	**−0.88**	−0.35	0.24
∆LA	−0.15	**0.82**	0.44	−0.04	−0.17	−0.17	**−0.92**	0.13	−0.09	0.49	**0.69**	**−0.70**	−0.55	0.12
FAT (kg)	0.62	−0.32	**0.70**	0.06	−0.66	**−0.69**	−0.14	−0.13	0.55	**−0.70**	0.10	**−0.81**	0.39	−0.42
FAT (%)	0.40	−0.35	**0.83**	0.11	−0.53	**−0.80**	−0.13	−0.13	0.37	**−0.76**	0.09	−0.38	0.50	**−0.69**
BMI	**0.78**	−0.48	0.16	−0.28	**−0.84**	−0.37	0.10	−0.25	**0.77**	−0.50	−0.08	**−0.73**	0.54	−0.21
MMS	**0.92**	−0.04	−0.37	−0.10	**−0.93**	0.28	0.06	0.11	0.55	**0.69**	−0.36	**−0.97**	0.16	0.10
BM	**0.99**	−0.13	−0.05	−0.06	**−0.99**	−0.05	0.00	0.02	**0.98**	−0.08	0.01	**−0.96**	0.16	0.06
FFM	**0.93**	−0.03	−0.35	−0.09	**−0.95**	0.27	0.05	0.06	**0.89**	0.07	0.10	**−0.97**	0.16	0.09
TBW	**0.93**	−0.04	−0.34	−0.11	**−0.95**	0.25	0.04	0.08	**0.89**	0.04	0.07	**−0.97**	0.16	0.10

Abbreviations: PPO—absolute value of maximum power; PPO_RV_—the relative value of maximum power; VEmax—maximum minute ventilation; BF—breathing frequency; HRmax—maximal heart rate; TTmax—maximum test duration; RER—respiratory exchange rate VCO_2_—volume CO_2_; ∆LA—delta blood lactate concentration during exercise; FAT (kg)—current body fat mass; FAT (%)—the percentage of body fat in total body weight; BMI—body mass index; MMS—skeletal muscle mass; BM—body mass; FFM—free fat mass; TBW—total body water.

**Table 5:** Final models after forwarding stepwise multivariate regression analysis for dependent variable VO_2_max on each training period from 1 to 4.

Model	Independent Variables	Beta	SE Beta	R	R^2^	Adjusted R^2^	SEE	ANOVA
Model 1	(Constant)	65.78 ***	0.69	0.91	0.82	0.72	2.06	0.025
	PC 2	1.35 *	0.41					
	PC 3	1.13	0.44					
	PC 4	1.52	0.64					
Model 2	(Constant)	69.56 ***	1.24	0.77	0.59	0.34	3.33	0.191
	PC 1	0.07	0.44					
	PC 2	0.77	0.81					
	PC 3	1.38	0.89					
	PC 4	−2.02	1.31					
Model 3	(Constant)	68.87 ***	0.94	0.80	0.64	0.54	2.96	0.028
	PC 2	−1.42 *	0.51					
	PC 1	−0.86	0.40					
Model 4	(Constant)	69.63 ***	0.31	0.87	0.76	0.73	0.88	0.005
	PC 2	−0.83 **	0.19					

Abbreviations: PC1, PC2, PC3, PC4—principal components; SEE = standard error of the estimation; ANOVA = *p*-value obtained from analysis of variance; * *p* ≤ 0.05; ** *p* ≤ 0.01; and *** *p* ≤ 0.001.

## Data Availability

Data are available on request due to privacy and ethical restrictions.
